# A New Constitutive Model Based on Taylor Series and Partial Derivatives for Predicting High-Temperature Flow Behavior of a Nickel-Based Superalloy

**DOI:** 10.3390/ma17143424

**Published:** 2024-07-11

**Authors:** Heping Deng, Xiaolong Wang, Jingyun Yang, Fanjiao Gongye, Shishan Li, Shixin Peng, Jiansheng Zhang, Guiqian Xiao, Jie Zhou

**Affiliations:** 1 Chongqing Key Laboratory of Advanced Mold Intelligent Manufacturing, College of Materials Science and Engineering, Chongqing University, Chongqing 400044, China; dhp623@163.com (H.D.);; 2 China National Erzhong Group Deyang Wanhang Die Forging Co., Ltd., Deyang 618013, China; 3 Chongqing For-Green Technology Co., Ltd., Chongqing 400044, China; 4 Chongqing Jiepin Technology Co., Ltd., Chongqing 400050, China

**Keywords:** nickel-based superalloy, constitutive model, Taylor series, partial derivatives, high-temperature behavior, flow stress prediction

## Abstract

Ni-based superalloys are widely used in aerospace applications. However, traditional constitutive equations often lack the necessary accuracy to predict their high-temperature behavior. A novel constitutive model, utilizing Taylor series expansions and partial derivatives, is proposed to predict the high-temperature flow behavior of a nickel-based superalloy. Hot compression tests were conducted at various strain rates (0.01 s^−1^, 0.1 s^−1^, 1 s^−1^, and 10 s^−1^) and temperatures (850 °C to 1200 °C) to gather comprehensive experimental data. The performance of the new model was evaluated against classical models, specifically the Arrhenius and Hensel–Spittel (HS) models, using metrics such as the correlation coefficient (R), root mean square error (RMSE), sum of squared errors (SSE), and sum of absolute errors (SAE). The key findings reveal that the new model achieves superior prediction accuracy with an R value of 0.9948 and significantly lower RMSE (22.5), SSE (16,356), and SAE (5561 MPa) compared to the Arrhenius and HS models. Additionally, the stability of the first-order partial derivative of logarithmic stress with respect to temperature (∂lnσ/∂T) indicates that the logarithmic stress–temperature relationship can be approximated by a linear function with minimal curvature, which is effectively described by a second-degree polynomial. Furthermore, the relationship between logarithmic stress and logarithmic strain rate ($\partial ln\sigma /\partial ln\dot{\varepsilon }$) is more precisely captured using a third-degree polynomial. The accuracy of the new model provides an analytical basis for finite element simulation software. This helps better control and optimize processes, thus improving manufacturing efficiency and product quality. This study enables the optimization of high-temperature forming processes for current superalloy products, especially in aerospace engineering and materials science. It also provides a reference for future research on constitutive models and high-temperature material behavior in various industrial applications.

## 1. Introduction

Superalloys, particularly those based on nickel, are widely used in aerospace, power generation, and various high-temperature settings due to their remarkable mechanical characteristics and ability to withstand harsh conditions [1,2,3]. These alloys are designed to endure high temperatures, corrosive environments, and significant mechanical stresses, making them ideal for gas turbine engines, aircraft structures, and nuclear reactors [4,5]. Among the nickel-based superalloys, GH4169, also known as Inconel 718, has gained considerable attention for its outstanding creep resistance, high strength, and exceptional corrosion resistance at high temperatures [6,7,8]. The material used in this paper is a typical GH4169 superalloy. The GH4169 superalloy is widely used in modern aero engines, gas turbines, and high-temperature components [9]. Its importance has led researchers to study the relationship between its plastic flow stress and deformation conditions. Constitutive models play a crucial role in understanding and predicting the flow behavior of materials under various processing conditions, such as temperature, strain rate, and strain [10]. A precise constitutive model is essential for optimizing the manufacturing processes of GH4169 superalloy components, such as hot forging, extrusion, and rolling [11]. By predicting flow stress under varying processing conditions, the model helps engineers design and control the manufacturing processes more effectively, resulting in enhanced product quality and lower production costs [12].

Classical constitutive models, such as the Arrhenius and Hensel–Spittel (HS) models, have been widely used to describe the high-temperature deformation behavior of various materials [13,14]. However, these models often lack the accuracy to effectively simulate the complex deformation behaviors of GH4169 under varying operational conditions. The Arrhenius model, based on the theory of thermally activated processes, presumes a linear relationship between the logarithm of flow stress and the logarithm of strain rate [15]. In contrast, the HS model is phenomenological, considering the influence of temperature, strain rate, and strain on flow stress through a multiplicative formula [16]. Despite the extensive use, the accuracy of these classical models in predicting the high-temperature flow behavior of the GH4169 superalloy has been questioned [17]. Numerous studies have highlighted discrepancies between predicted and experimental flow stress values, particularly at elevated strain rates and temperatures [18,19]. Lin et al. [20] developed models considering the coupled effects of deformation temperatures and strain rate. Xiao et al. [21] established a new physic-based constitutive model for GH4169 based on experimental results, considering the effects of dislocation movement. Zhou et al. [22] developed constitutive models for GH4169 that predict flow stresses considering the coupled effects of deformation parameters during work hardening-dynamic recovery (DRV) and dynamic recrystallization (DRX) periods. 

In recent years, advanced mathematical techniques like artificial neural networks (ANNs) and support vector regression (SVR) have been applied to develop constitutive models for various materials [23,24]. These methods have shown promising results in capturing the complex nonlinear relationships between flow stress and deformation variables. For instance, Zhu et al. [25] developed an ANN-based constitutive model for the GH4169 superalloy, which demonstrated improved accuracy compared to classical models. Similarly, Wen et al. [26] employed SVR to predict the flow behavior of the GH4169 superalloy, achieving high prediction accuracy. Zheng et al. [27] established a constitutive model for GH4169 based on the Arrhenius equation and optimized the model parameters using a backpropagation artificial neural network (BP-ANN). Gu et al. [28] constructed a data-driven constitutive model based on experimental data to describe the deformation behavior of the GH4169 superalloy. Wen et al. [29] proposed a two-stage deep learning-based constitutive model framework for GH4169 that incorporates a deformation-induced temperature rise and its influence on flow stress, comparing it favorably to the traditional Arrhenius-type model. However, the interpretability of these models remains a challenge, and their application in the GH4169 superalloy is somewhat limited. The models presented in these papers are difficult to apply to numerical simulation software, thus highlighting the need to develop a new model with a mathematical expression that can be used in numerical simulation software.

Recent advancements have highlighted the potential of higher-order mathematical approaches to improve the accuracy of constitutive models. The use of Taylor series expansions and partial derivatives in constitutive modeling has attracted considerable attention. These approaches offer a promising path for developing more accurate predictive models by capturing subtle variations in material behavior under different conditions [30]. In the context of the GH4169 superalloy, limited research has been conducted on the application of Taylor series expansions and partial derivatives for constitutive modeling. This study aims to address these gaps by proposing a novel constitutive model that leverages Taylor series expansions and partial derivatives to predict the high-temperature flow behavior of GH4169 superalloy. By conducting comprehensive hot compression tests and evaluating the new model’s performance against classical models, this study seeks to provide a more accurate and reliable tool for predicting the flow stress of GH4169 under various strain rates and temperatures. The development of this model will advance constitutive modeling techniques and optimize manufacturing processes for GH4169 superalloy components in aerospace and other high-temperature industries.

## 2. Materials and Experiments

### 2.1. Material

**Figure 1:**
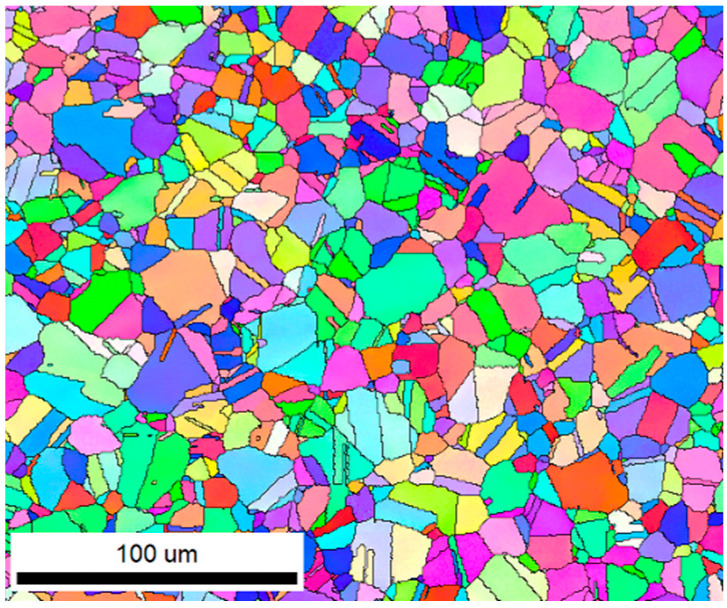
Original microstructure of the wrought GH4169 superalloy.

The experimental material used in this study is the wrought GH4169 superalloy, supplied by Deyang Wanhang Die Forging Co., Ltd. in Deyang, China, and its nominal chemical composition is listed in Table 1. The alloying elements in GH4169 are Ni, Fe, Cr, Nb, and Mo. The original microstructure of the wrought GH4169 alloy is shown in Figure 1.

### 2.2. Experimental Procedures and Results

**Figure 2:**
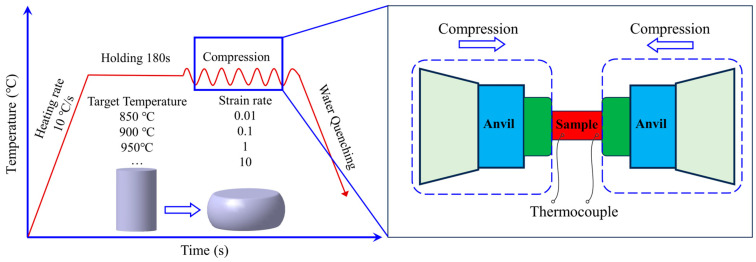
The thermomechanical process diagram.

Hot forming is a critical technology for superalloys in the aerospace industry. Elevated temperatures during hot forming enable significant plastic deformation of metallic alloys that exhibit limited ductility at room temperature. Hot forging processes for superalloys must achieve optimal high-temperature mechanical properties to meet performance specifications under extreme thermal conditions [31]. To investigate the hot forming of the GH4169 superalloy in detail, eight temperatures (850 °C, 900 °C, 950 °C, 1000 °C, 1050 °C, 1100 °C, 1150 °C, and 1200 °C) and four strain rates (0.01^−1^, 0.1^−1^, 1^−1^, and 10^−1^) were selected. Consequently, 32 cylindrical samples were processed from the wrought rod. Hot deformation samples with a height of 9 mm and a diameter of 6 mm were prepared using wire electrodischarge machining. The samples were individually heated to the target temperature inside the Gleeble-3500 thermal compression testing machine (Data Sciences International, Inc., St. Paul, MN, USA) at a rate of 10 °C/s. Subsequently, they were isothermally compressed at different strain rates (0.01^−1^, 0.1^−1^, 1^−1^, and 10^−1^) to achieve a deformation of 60%. The isothermal compression process and corresponding experimental parameters are shown in Figure 2 and Table 2, respectively. After each sample completed the isothermal compression test, it was quenched in water. The flow stress–strain curves were automatically recorded from the load-displacement data during the isothermal compression experiments.

**Figure 3:**
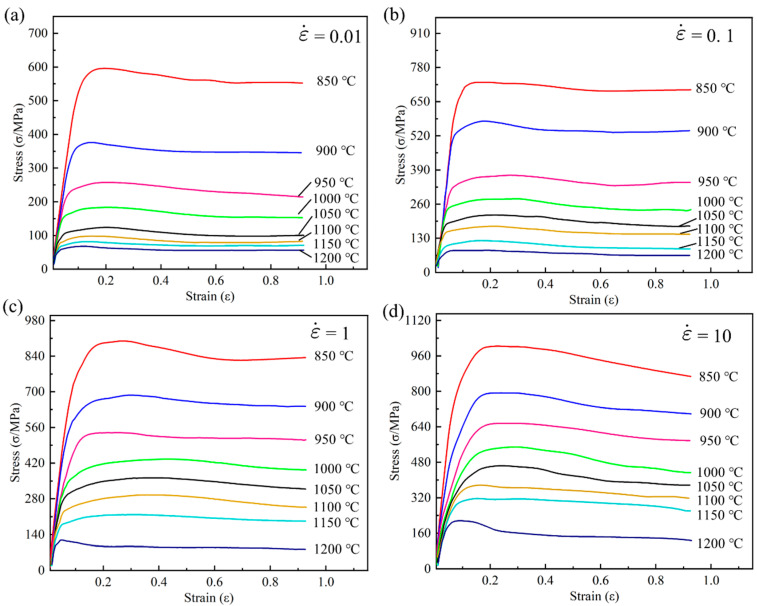
Flow stress–strain curves of the superalloy during hot compression at different strain rates: (**a**) 0.01 s^−1^; (**b**) 0.1 s^−1^; (**c**) 1 s^−1^; and (**d**) 10 s^−1^.

Figure 3 shows the stress–strain curves at different strain rates and deformation temperatures. At the same strain rate, the stress gradually decreases with increasing deformation temperature, and the peak stress also decreases as the temperature rises. For a given strain rate at a specific temperature, the stress sharply increases with strain during the initial stages of deformation until it reaches its maximum value. This increase is primarily attributed to work hardening. As deformation continues, dislocation slip occurs, leading to a sharp increase in dislocation density within the grains, resulting in dislocation interactions. In the later stages, stress decreases with increasing strain due to dynamic recovery and recrystallization. When the deformation reaches a certain level, the stored energy becomes the driving force for recrystallization, initiating dynamic recrystallization. The occurrence of dynamic recrystallization leads to the disappearance of internal dislocations, reducing the material’s deformation resistance. As deformation progresses, the recrystallization continues until it is complete. After complete internal dynamic recrystallization, the flow stress reaches a steady-state stage. Furthermore, the non-monotonic behavior in the plot at 1200 °C in Figure 3d (depicted by the blue curve at the bottom) shows an initial increase in stress with strain, reaching a peak stress at a relatively low strain value (around 0.05). After reaching this peak, the stress decreases significantly, exhibiting a softening behavior. The stress continues to drop as the strain increases, indicating dynamic recrystallization or flow softening mechanisms at this elevated temperature. This behavior suggests that at 1200 °C, the material undergoes significant softening after the initial hardening, likely due to the thermal activation of mechanisms that reduce dislocation density or promote grain boundary sliding. To investigate the constitutive behavior of the superalloy, the flow stress curve was discretized into ten equal intervals, covering a strain range from 0.04 to 0.9. The stress values in Table 3 were obtained by linear interpolation of the flow curves shown in Figure 3. The colors in Table 3 transition from light to dark, from white to red, where a deeper red background indicates higher stress values. Analyzing the data for a specific strain shows that, at the same temperature, the stress value increases with the strain rate. Conversely, at the same strain rate, the stress value gradually decreases with an increase in temperature. Comparing these 10 sets of data clearly shows that they exhibit the same trend.

## 3. Classical Constitutive Model

For accurate numerical simulations, a reliable flow curve prediction model must be established. The flow curve describes the relationship between the flow stress and strain of a material at different temperatures and strain rates. A constitutive model is a mathematical expression used to fit the experimental data of the flow curve. In this paper, a comparison is made between two commonly used constitutive models, the Arrhenius model and the Hensel–Spittel (HS) model, before proposing a new phenomenological constitutive model. The Arrhenius model is based on activation energy theory, which assumes that the logarithm of flow stress has a linear relationship with temperature and strain rate. The Hensel–Spittel (HS) model is an empirical model that assumes flow stress has a linear relationship with a power function of temperature and strain rate.

### 3.1. Arrhenius Model

The Arrhenius model was first proposed by Sellars and Tegart [32]. This widely used constitutive model describes the relationship between flow stress, temperature, and strain rate during high-temperature plastic deformation of alloys. It assumes that flow stress is a hyperbolic sine function of temperature T and deformation activation energy Q, with its mathematical expression outlined as follows:

(1) $$\dot{\varepsilon }=\left\{\begin{array}{c} A{\left[sinh\left(\alpha \sigma \right)\right]}^{n}exp\left(-Q/RT\right)\;for\;all\;\sigma \\ A_{1}exp\left(\beta \sigma \right)exp\left(-Q/RT\right)\;for\;\alpha \sigma >1.2\\ A_{2}\sigma^{n}exp\left(-Q/RT\right)\;for\;\alpha \sigma <0.8 \end{array}\right.$$

where $\dot{\varepsilon }$ is the strain rate of the material (s^−1^), σ is flow stress of the material (MPa), Q is the activation energy of thermal deformation (J·mol^−1^), R is the universal gas constant (8.314 J·K^−1^·mol^−1^), T is the thermodynamic temperature of the material (K), α is the stress level parameter, A is the structure factor (s^−1^), n is the stress index, and A, α, n, β (β=αn) and Q are the material parameters.

Taking the logarithm of the Equation (1):

(2) $$\ln\dot{\varepsilon }=\left\{\begin{array}{c} \ln A+n\ln\left[sinh\left(\alpha \sigma \right)\right]-\;\left(\frac{Q}{RT}\right)\;\;for\;all\;\sigma \\ \ln A_{1}+\beta \sigma -\frac{Q}{RT}\;for\;\alpha \sigma >1.2\\ \ln A_{2}+n\ln\sigma -\frac{Q}{RT}\;for\;\alpha \sigma <0.8 \end{array}\right.$$

**Figure 4:**
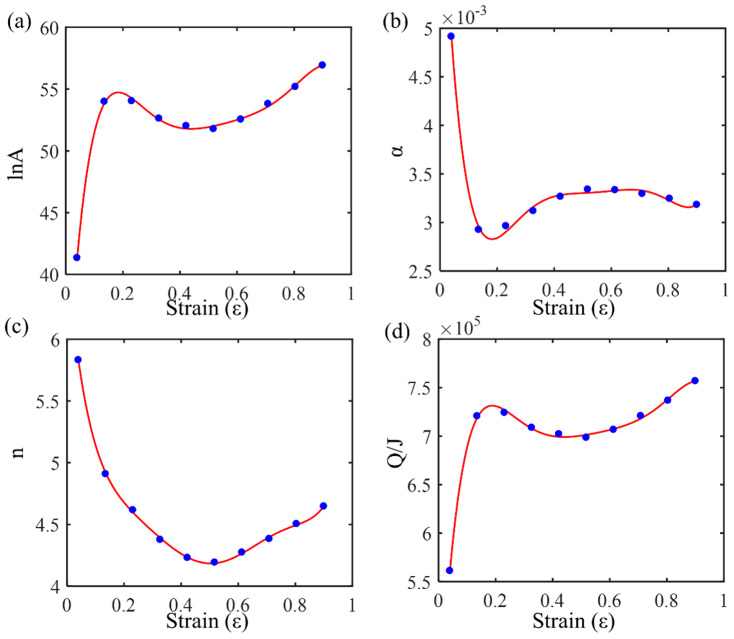
Polynomial fitting curves corresponding to different strains: (**a**) lnA-*ε*; (**b**) *α*-*ε*; (**c**) *n*-*ε*; and (**d**) *Q*-*ε*.

Multivariate nonlinear regression based on Equation (2) was performed with the flow stress data to solve for the material parameters lnA, α, n and Q at different strain levels (Table 3). Then, the curves of lnA-ε, α-ε, n-ε, and Q-ε (as shown in Figure 4) were fitted with polynomials to obtain the constitutive equation with strain compensation.

As shown in Figure 4a, the regression values of lnA start at around 40 and rise sharply to around 55 at a strain level of 0.2. After reaching this peak, the values decrease slightly and then stabilize around 55 as the strain increases further, exhibiting a gentle upward trend toward a strain level of 0.9. As shown in Figure 4b, the regression values of α start at 0.005 and decrease rapidly to about 0.003 at a strain level of 0.2. After this initial drop, the values gradually increase, showing a slight upward trend toward a strain level of 0.9. As shown in Figure 4c, the regression values of n start at about 6 and decrease significantly to about 4.5 at a strain level of 0.2. After this initial drop, the values stabilize around 4.5, showing a gentle downward trend as the strain increases further. As shown in Figure 4d, the regression values of Q start at about 5.5 and rise sharply to about 7 at a strain level of 0.2. After reaching this peak, the values decrease slightly and then stabilize around 7 as the strain increases further, exhibiting a gentle upward trend toward a strain level of 0.9.

A sixth-order polynomial is used to fit these data, thereby obtaining parameter-containing expressions as shown in Equation (3). The polynomial order is determined by the regression accuracy, but a higher order may cause overfitting.

(3) $$\left\{\begin{array}{c} \ln A=-2465.7\varepsilon^{6}+8,147,348\varepsilon^{5}-10,782\varepsilon^{4}+7255.07\varepsilon^{3}-2560\varepsilon^{2}+430\varepsilon +27.83\\ \alpha =0.4447\varepsilon^{6}-1.4428\varepsilon^{5}+1.8662\varepsilon^{4}-1.2216\varepsilon^{3}+0.4198\varepsilon^{2}-0.069\varepsilon +0.007\\ n=219.57\varepsilon^{6}-668.06\varepsilon^{5}+791.78\varepsilon^{4}-464.37\varepsilon^{3}+145.74\varepsilon^{2}-25.8\varepsilon +6.66\\ Q=-309,460,450\varepsilon^{6}+101,761,981\varepsilon^{5}-133,986,577\varepsilon^{4}+89,771,783\varepsilon^{3}-31,638,223\varepsilon^{2}+5,346,899\varepsilon +392,766 \end{array}\right.$$

**Figure 5:**
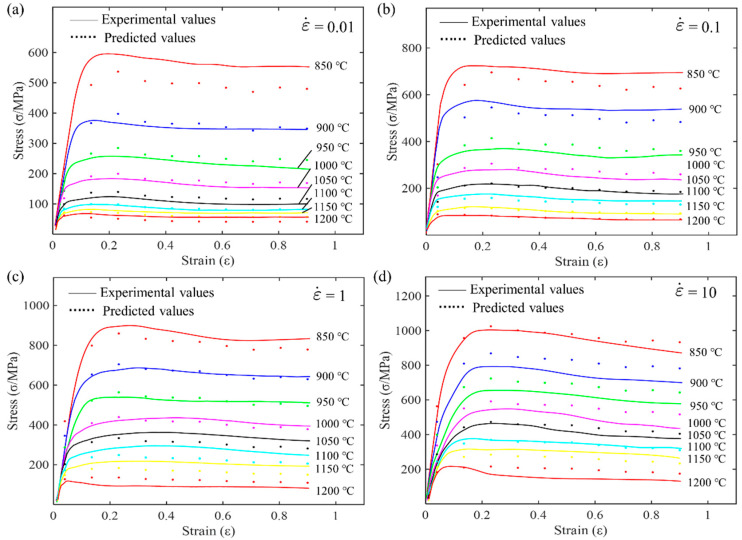
Comparison of flow stress predicted by the Arrhenius model with experimental values at different strain rates: (**a**) 0.01 s^−1^; (**b**) 0.1 s^−1^; (**c**) 1 s^−1^; and (**d**) 10 s^−1^.

By substituting Equation (3) into Equation (1), the Arrhenius constitutive equation for GH4169 alloy can be obtained. To visually observe the prediction accuracy of the model, the experimental data and predicted data from the Arrhenius constitutive equation are plotted on the same graph for comparison, as shown in Figure 5.

Comparing the curves at four different strain rates reveals that as the strain rate increases, the material’s flow stress level increases significantly. This indicates the strain rate sensitivity of the material. At high strain rates, dislocation movement is impeded, resulting in significant material strengthening. At a given strain rate, as the temperature increases, the flow stress gradually decreases. At high temperatures, thermal activation processes intensify, dislocation motion resistance decreases, and the material softens. The flow stress peaks at 850 °C and is lowest at 1200 °C. In the low strain range, the model’s predicted values slightly deviate from the experimental values. It is speculated that in the small strain stage, complex microstructural evolution behaviors such as strain hardening and dynamic recovery may occur, which the Arrhenius model cannot fully describe. At a high strain rate of 10 s^−1^, the model’s deviation slightly increases. This suggests that the Arrhenius model is more suitable for low strain rate conditions. At high strain rates, the introduction of other dynamic softening factors may be necessary. Overall, the Arrhenius model can describe high-temperature rheology to some extent, but its quantitative accuracy requires further improvement.

### 3.2. Hensel–Spittel Constitutive Model

The Hensel–Spittel (HS) constitutive model accurately describes the relationship between flow stress, strain, strain rate, and temperature during the thermal deformation of metallic materials [33]. By selecting appropriate model parameters, the HS constitutive model can predict the mechanical behavior of various metallic materials under different processing parameters, providing an important basis for optimizing and controlling the hot forming process [34]. Furthermore, the HS constitutive model has gained extensive application in hot forming numerical simulations due to its simple mathematical form and easily accessible material parameters [35,36,37,38]. Renowned commercial finite element software like Forge NxT has incorporated this model into their material constitutive libraries, showcasing its universality and effectiveness in the engineering field, as shown in Equation (4):

(4) $$\sigma =Ae^{m_{1}T}\varepsilon^{m_{2}}{\dot{\varepsilon }}^{m_{3}}e^{m_{4}/\varepsilon }(1+\varepsilon {)}^{m_{5}T}e^{m_{6}\varepsilon }{\dot{\varepsilon }}^{m_{7}T}T^{m_{8}}$$

where ε, σ, $\dot{\varepsilon }$, and T represent strain, flow stress, strain rate, and temperature, respectively, and A and m_1_~m_8_ are material constants. Taking the natural logarithm of Equation (4):

(5) $$\begin{array}{cc} \ln\sigma = & \ln A+m_{1}T+m_{2}\ln\varepsilon +m_{3}\ln\dot{\varepsilon }+m_{4}/\varepsilon +\\ & m_{5}T\ln\left(1+\varepsilon \right)+m_{6}\varepsilon +m_{7}T\ln\dot{\varepsilon }+m_{8}\ln T \end{array}$$

**Figure 6:**
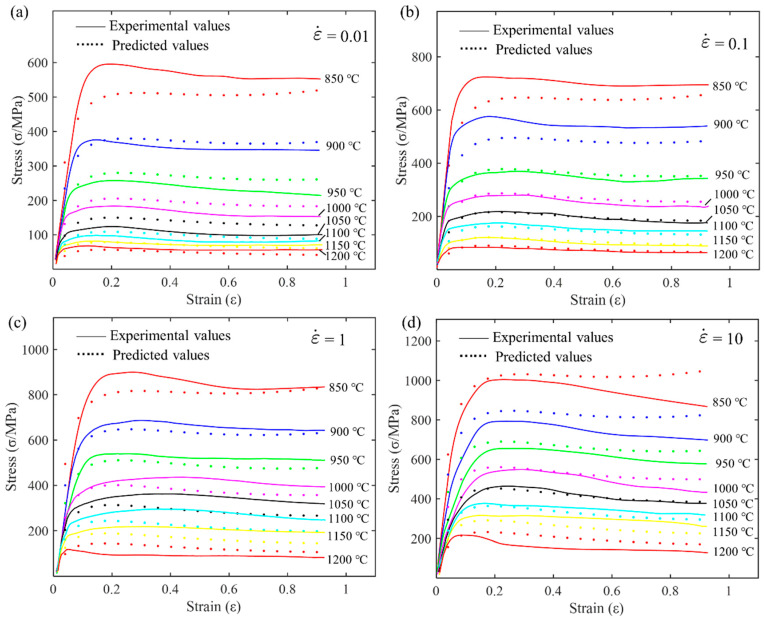
Comparison of predicted and experimental flow stresses for the HS model at different strain rates: (**a**) 0.01 s^−1^; (**b**) 0.1 s^−1^; (**c**) 1 s^−1^; and (**d**) 10 s^−1^.

There is a linear relationship between lnσ and T, lnε, ln$\dot{\varepsilon }$, 1/ε, Tln(1+ε), ε, Tln$\dot{\varepsilon }$, and Tlnε. Therefore, solving for the material constants in Equation (5) is a typical multivariate linear regression problem. Similarly, the strain is divided into ten equal intervals between 0.04 and 0.9. Then, by interpolating the original compression data (as shown in Figure 3), the flow stress at each strain level can be obtained. Using MATLAB software (Version R2016b) to solve for the parameters, the multivariate linear regression results for the material parameters are listed in Table 4. The comparison of the HS model’s predicted flow stress with the experimental flow stress is shown in Figure 6.

### 3.3. Evaluation of the Applicability of Traditional Constitutive Models

To visually evaluate the predictive capability of the Arrhenius model and the Hensel–Spittel model for the high-temperature flow behavior of wrought GH4169 alloy, their calculated values were compared with experimental values. Figure 5 and Figure 6, respectively, show the comparison of the predicted and experimental flow stress values for the Arrhenius model and the HS model. It can be seen that the error is larger at lower temperatures of 850 °C, 900 °C, and 950 °C. As shown in Figure 6, the predictive accuracy of the HS constitutive equation is lower at lower temperatures (850 °C, 900 °C, and 950 °C), while it is higher in the high-temperature range (1000 °C, 1050 °C, 1100 °C, 1150 °C, and 1200 °C).

In conclusion, the traditional Arrhenius and Hensel–Spittel models can describe the high-temperature flow behavior of the wrought GH4169 superalloy to some extent. Overall, the predictive accuracy of the HS model is higher than that of the Arrhenius model, but both models still face challenges in further improving their predictive accuracy. The main reasons for the prediction deviations are that neither model considers the dependence of flow stress on strain, whereas the flow stress of the GH4169 superalloy actually changes significantly with strain. Additionally, traditional constitutive models lack consideration of the interaction between different deformation parameters (such as temperature, strain rate, and strain). Therefore, it is necessary to develop new constitutive models to more accurately describe the high-temperature flow characteristics of the wrought GH4169 superalloy.

## 4. Development of a New Constitutive Model

The Arrhenius model highlights that the key to constructing the constitutive equation under specific strain is determining the mathematical relationship among stress, strain rate, and temperature. However, the Arrhenius model itself is an implicit equation, making it relatively complex to solve in engineering applications. Additionally, the fitting accuracy of this model for the stress–strain values of the GH4169 alloy requires improvement. Theoretically, the relationship between stress, strain rate, and temperature can be represented as $\sigma =f\left(\varepsilon ,\dot{\varepsilon },T\right)$. However, due to the significant nonlinearity among these three variables, this study did not directly use this functional form. Before constructing a new constitutive model, the influence of strain rate and temperature on stress was investigated. To this end, experimental data at low strain (0.136), medium strain (0.518), and high strain (0.9) levels from Table 3 were analyzed. To reveal the intrinsic relationship between logarithmic stress, logarithmic strain rate, and temperature, the partial derivatives of logarithmic stress with respect to temperature and logarithmic strain rate were calculated using numerical discretization methods, such as finite differences. Common numerical discretization methods include forward difference, backward difference, and central difference. To ensure computational accuracy and stability, forward and backward differences were used at boundary points, while central differences were used at internal nodes.

### 4.1. Mathematical Principles

Partial derivatives are an important concept in multivariable calculus, indicating the rate of change in a function in the direction of a specific variable. For a two-variable function f(x,y), the partial derivative in the x-direction is defined as follows [39]:

(6) $$\frac{\partial f(x,y)}{\partial x}=lim\left[\frac{f\left(x+∆x,y\right)-f(x,y)}{∆x}\right]$$

Similarly, the partial derivative of the function in the y-direction is defined as follows:

(7) $$\frac{\partial f(x,y)}{\partial y}=lim\left[\frac{f\left(x,y+∆y\right)-f(x,y)}{∆y}\right]$$

In practical calculations of partial derivatives, numerical discretization methods are commonly employed, approximating derivatives using finite differences. Similarly, second-order and higher-order derivatives can be obtained through the recursive application of first-order finite differences. To ensure accuracy when calculating derivatives at boundary points, one-sided difference schemes are typically used, whereas central difference schemes are often employed at internal nodes to balance accuracy and stability.

The Taylor series is an important local expansion of functions used to approximate complex nonlinear functions. If a function f(x) has continuous derivatives up to the nth order at the point $x_{0}$, its Taylor expansion is given as follows [40]:

(8) $$f\left(x\right)=f\left(x_{0}\right)+\left(x-x_{0}\right)f^{\prime }\left(x_{0}\right)+{\left(x-x_{0}\right)}^{2}(f^{\prime \prime }\left(x_{0}\right))\;/2!+\cdots \;=\sum_{n=0}^{\infty }\;\frac{f^{\left(n\right)}\left(x_{0}\right)}{n!}{\left(x-x_{0}\right)}^{n}$$

Nickel-based superalloys exhibit significant nonlinear mechanical behavior under high strain rates and high-temperature conditions, with complex nonlinear coupling effects between stress levels, strain rates, and temperatures. Experimental data at strain levels of 0.136, 0.518, and 0.9 from Table 3 were selected for analysis. To quantitatively describe the functional relationship between logarithmic stress, temperature, and logarithmic strain rate, the partial derivatives of logarithmic stress with respect to temperature and logarithmic strain rate were calculated using discrete formulas. The discrete formulas include forward difference, backward difference, and central difference. Forward difference and backward difference were used at the initial and final boundaries, respectively, while central difference was used in the intermediate region. Based on the discrete difference method, the nth-order partial derivatives of logarithmic stress with respect to temperature and logarithmic strain rate at any point on the experimental stress–strain curve can be calculated using Formula (9) as the central difference.

(9) $$\left\{\begin{array}{c} {\left.\frac{\partial^{n}ln\sigma }{\partial T^{n}}\right|}_{i,j}=\frac{{\left.\frac{\partial^{n-1}ln\sigma }{\partial T^{n-1}}\right|}_{i,j+1}-{\left.\frac{\partial^{n-1}ln\sigma }{\partial T^{n-1}}\right|}_{i,j-1}}{T_{i,j+1}-T_{i,j-1}}\\ {\left.\frac{\partial^{n}ln\sigma }{\partial {\left(ln\dot{\varepsilon }\right)}^{n}}\right|}_{i,j}=\frac{{\left.\frac{\partial^{n-1}ln\sigma }{\partial {\left(ln\dot{\varepsilon }\right)}^{n-1}}\right|}_{i+1,j}-{\left.\frac{\partial^{n-1}ln\sigma }{\partial {\left(ln\dot{\varepsilon }\right)}^{n-1}}\right|}_{i-1,j}}{ln{\dot{\varepsilon }}_{i+1,j}-ln{\dot{\varepsilon }}_{i-1,j}} \end{array}\right.$$

where n represents the order of the partial derivative, and i and j represent the indices of strain rate and temperature, respectively. For example, when i = 1 and j = 1, ln$\sigma_{ij}$ denotes the logarithmic stress at a strain rate of 0.01 s^−1^ and a temperature of 850 °C. The maximum values of i and j correspond to the number of strain rates and temperatures, respectively.

When i = 1 and j = 1, Formula (9) transforms into the backward difference Formula (10).

(10) $$\left\{\begin{array}{c} {\left.\frac{\partial^{n}ln\sigma }{\partial {ln\dot{\varepsilon }}^{n}}\right|}_{i,j}=\frac{{\left.\frac{\partial^{n-1}ln\sigma }{\partial {ln\dot{\varepsilon }}^{n-1}}\right|}_{i+1,j}-{\left.\;\frac{\partial^{n-1}ln\sigma }{\partial {ln\dot{\varepsilon }}^{n-1}}\right|}_{i,j}}{ln{\dot{\varepsilon }}_{i+1,j}-ln{\dot{\varepsilon }}_{i,j}}\\ {\left.\frac{\partial^{n}ln\sigma }{\partial T^{n}}\right|}_{i,j}=\frac{{\left.\frac{\partial^{n-1}ln\sigma }{\partial T^{n-1}}\right|}_{i,j+1}-{\left.\;\frac{\partial^{n-1}ln\sigma }{\partial T^{n-1}}\right|}_{i,j}}{T_{i,j+1}-T_{i,j}} \end{array}\right.$$

When i = 4 and j = 8, Formula (9) transforms into the forward difference Formula (11).

(11) $$\left\{\begin{array}{c} {\left.\frac{\partial^{n}ln\sigma }{\partial {ln\dot{\varepsilon }}^{n}}\right|}_{i,j}=\frac{{\left.\frac{\partial^{n-1}ln\sigma }{\partial {ln\dot{\varepsilon }}^{n-1}}\right|}_{i,j}-{\left.\frac{\partial^{n-1}ln\sigma }{\partial {ln\dot{\varepsilon }}^{n-1}}\right|}_{i-1,j}}{ln{\dot{\varepsilon }}_{i,j}-{ln\dot{\varepsilon }}_{i-1,j}}\\ {\left.\frac{\partial^{n}ln\sigma }{\partial T^{n}}\right|}_{i,j}=\frac{{\left.\frac{\partial^{n-1}ln\sigma }{\partial T^{n-1}}\right|}_{i,j}-{\left.\;\frac{\partial^{n-1}ln\sigma }{\partial T^{n-1}}\right|}_{i,j-1}}{T_{i,j}-T_{i,j-1}} \end{array}\right.$$

**Figure 7:**
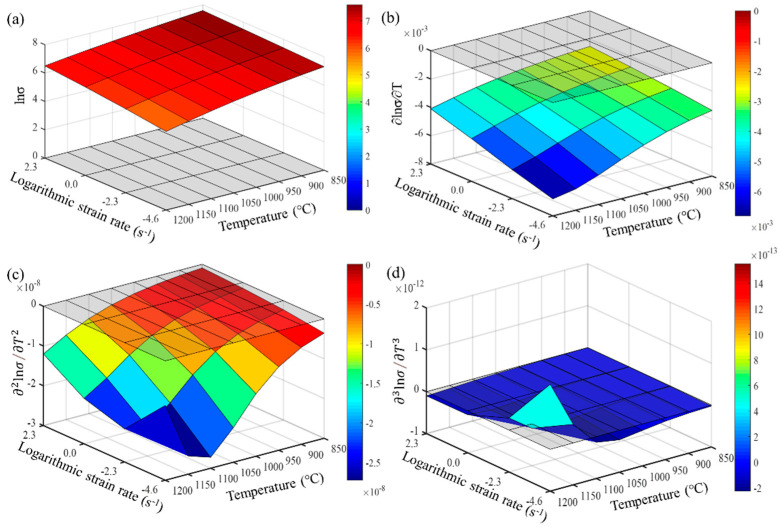
Different orders of partial derivatives of logarithmic stress with respect to temperature at a low strain level (0.136): (**a**) zeroth order; (**b**) first order; (**c**) second order; and (**d**) third order.

**Figure 8:**
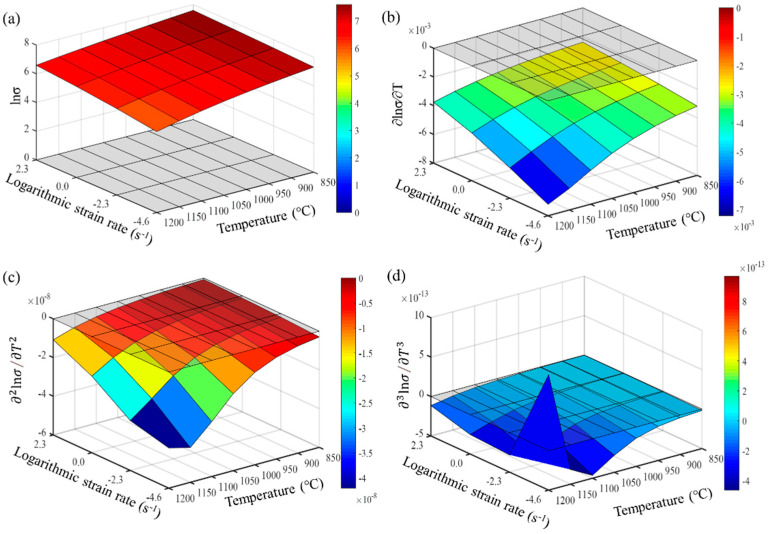
Different orders of partial derivatives of logarithmic stress with respect to temperature at a medium strain level (0.518): (**a**) zeroth order; (**b**) first order; (**c**) second order; and (**d**) third order.

**Figure 9:**
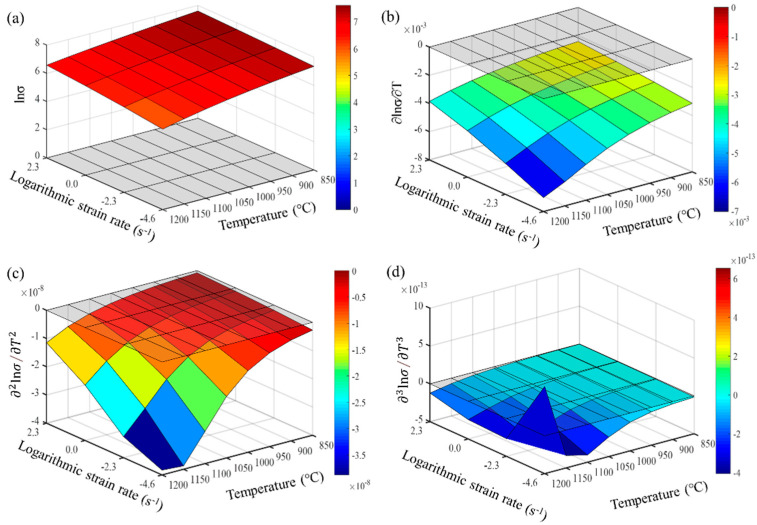
Different orders of partial derivatives of logarithmic stress with respect to temperature at a high strain level (0.9): (**a**) zeroth order; (**b**) first order; (**c**) second order; and (**d**) third order.

From Formulas (9)–(11), it is evident that the accuracy of calculating the partial derivatives of stress is closely related to the step size of the finite differences. The denser the experimental points for temperature and strain rate, the closer the numerical calculation results are to the actual partial derivatives. Maximizing the number of experimental data points is crucial for accurately describing the constitutive behavior of the wrought GH4169 superalloy. The first, second, and third-order partial derivatives of logarithmic stress with respect to temperature and logarithmic strain rate were calculated at strain levels of 0.136, 0.518, and 0.9. The relevant results are shown in Figure 7, Figure 8 and Figure 9, respectively. For the three strain levels, logarithmic stress decreases with increasing deformation temperature (as shown in Figure 7a, Figure 8a and Figure 9a). This indicates that, at a given strain level, higher strain rates lead to higher stress values. At a specific strain level, the first-order partial derivatives of logarithmic stress with respect to temperature (∂lnσ/∂T) show little difference at different temperatures, with a magnitude of about 0.001, as shown in Figure 7b, Figure 8b and Figure 9b. This indicates that the rate of change in logarithmic stress with temperature (i.e., the slope of the logarithmic stress–temperature curve) remains relatively stable within the studied temperature range. The second-order partial derivatives ($\partial^{2}ln\sigma /\partial T^{2}$) and third-order partial derivatives ($\partial^{3}ln\sigma /\partial T^{3}$) of logarithmic stress with respect to temperature are close to zero at all strain levels, with smaller magnitudes, as shown in Figure 7c,d, Figure 8c,d and Figure 9c,d.

From the Taylor series Formula (8), it can be seen that if the Taylor series expansion of f(x) near the point $x_{0}$ is written out and the fourth and higher-order terms are ignored, the following can be obtained:

(12) $$f\left(x\right)=f\left(x_{0}\right)+\left(x-x_{0}\right)f^{\prime }\left(x_{0}\right)+\frac{{\left(x-x_{0}\right)}^{2}}{2!}f^{\prime \prime }\left(x_{0}\right)+\frac{{\left(x-x_{0}\right)}^{3}}{3!}f^{\prime \prime \prime }\left(x_{0}\right)$$

The constant term $f(x_{0})$, which represents the value of the function at $x_{0}$, determines the vertical position of the Taylor series expansion but does not affect the shape of the function. The first-order term is $\left(x-x_{0})f^{\prime }(x_{0}\right)$, which is a linear function with a slope given by the first derivative $f^{\prime }(x_{0})$. When x is close to $x_{0}$, this term causes the function to exhibit approximately linear behavior near $x_{0}$. The second-order term ${\left(x-x_{0}\right)}^{2}f^{\prime \prime }(x_{0})/2!$ is a quadratic function with a coefficient determined by the second derivative $f^{\prime \prime }(x_{0})$. If $f^{\prime \prime }(x_{0})$ is not zero, this term introduces curvature, causing the function f(x) to deviate from linearity. However, if $f^{\prime \prime }(x_{0})$ is close to zero, the contribution of this term becomes very small, and the function f(x) still exhibits approximately linear behavior near $x_{0}$. The third-order term ${\left(x-x_{0}\right)}^{3}f^{\prime \prime \prime }(x_{0})/3!$ is a cubic function with a coefficient determined by the third derivative $f^{\prime \prime \prime }(x_{0})$. Similar to the second-order term, if $f^{\prime \prime \prime }(x_{0})$ is close to zero, the contribution of this term also becomes very small. This implies that even if the function f(x) exhibits slight nonlinearity near $x_{0}$, its rate of curvature change is very small, and it can still be well approximated by a linear function overall.

The first-order partial derivative of logarithmic stress with respect to temperature (∂lnσ/∂T) is relatively stable within the studied temperature range. This indicates that the logarithmic stress–temperature curve can be well approximated by a straight line with a constant slope within this temperature range. The second-order partial derivative ($\partial^{2}ln\sigma /\partial T^{2}$) and third-order partial derivative ($\partial^{3}ln\sigma /\partial T^{3}$) of logarithmic stress with respect to temperature are very close to zero within the studied temperature range. This suggests that the logarithmic stress–temperature curve has almost no curvature or rate of curvature change within this temperature range, exhibiting characteristics very close to linearity. However, to fit the experimental curve more precisely, higher-order partial derivatives are considered. Therefore, the relationship between logarithmic stress lnσ and temperature T within the studied temperature range can be described with high accuracy by a second-order function.

The importance of this conclusion is that it provides a simple and effective method for describing the law of variation of material flow stress with temperature. By analyzing the higher-order partial derivatives of logarithmic stress, the linearity of the lnσ-T relationship can be directly judged, thereby selecting an appropriate mathematical model to fit the experimental data. This not only greatly simplifies the process of establishing the constitutive model but also improves the computational efficiency and predictive capability of the model. It provides a theoretical foundation for establishing efficient and practical material constitutive models. This method is not only applicable to the high-temperature alloy studied in this paper but can also be extended to other types of materials, offering broad application prospects.

**Figure 10:**
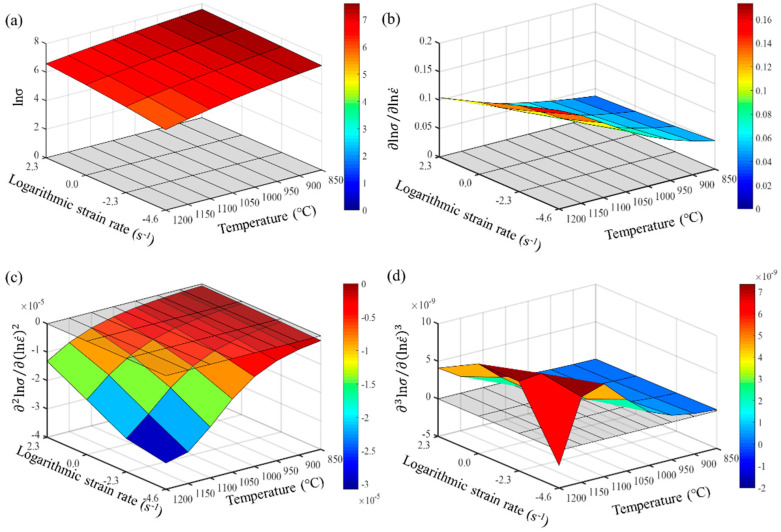
Different orders of partial derivatives of logarithmic stress with respect to logarithmic strain rate at a low strain level (0.136): (**a**) zeroth order; (**b**) first order; (**c**) second order; and (**d**) third order.

**Figure 11:**
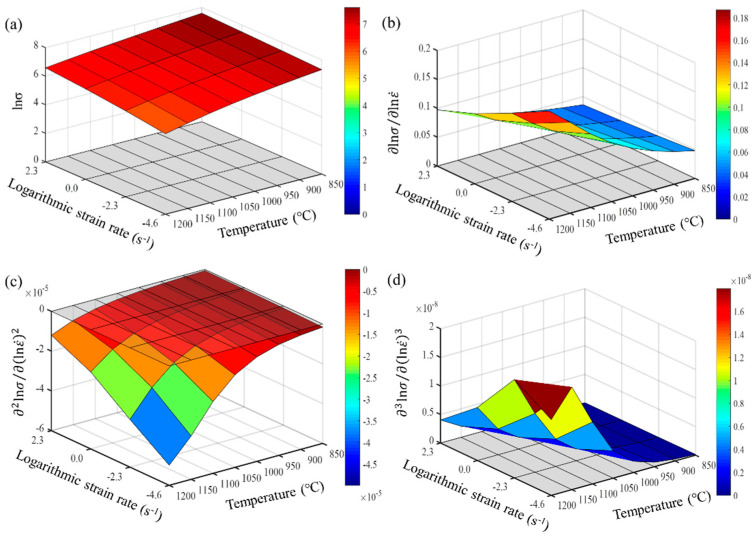
Different orders of partial derivatives of logarithmic stress with respect to logarithmic strain rate at a medium strain level (0.518): (**a**) zeroth order; (**b**) first order; (**c**) second order; and (**d**) third order.

**Figure 12:**
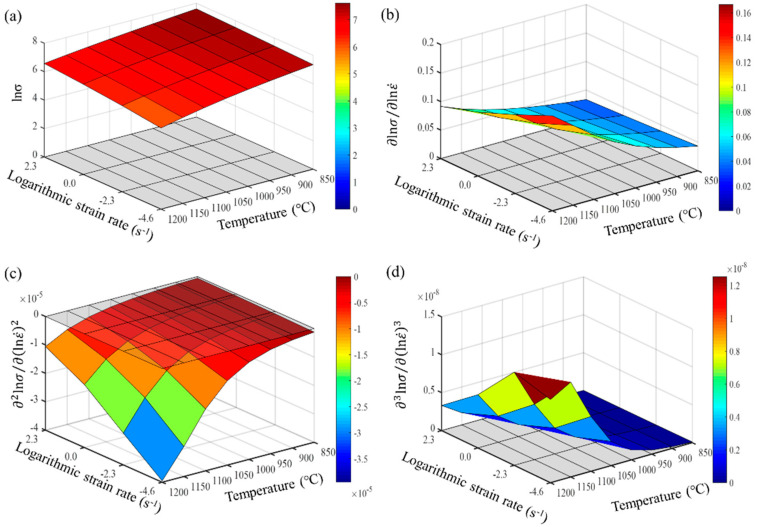
Different orders of partial derivatives of logarithmic stress with respect to logarithmic strain rate at a high strain level (0.9): (**a**) zeroth order; (**b**) first order; (**c**) second order; and (**d**) third order.

For the studied strain levels (low strain ε = 0.136, medium strain ε = 0.518, and high strain ε = 0.9), logarithmic stress increases with the increase in logarithmic strain rate (as shown in Figure 10a, Figure 11a and Figure 12a). The first-order partial derivative of logarithmic stress with respect to logarithmic strain rate ($\partial ln\sigma /\partial ln\dot{\varepsilon }$) varies significantly at all strain levels (as shown in Figure 10b, Figure 11b and Figure 12b), and the absolute value of the first-order partial derivative increases with the increase in temperature and logarithmic strain rate. This indicates that the impact of strain rate on flow stress becomes more significant with an increase in temperature and strain rate. In other words, the material is more sensitive to strain rate under high temperature and high strain rate conditions. Furthermore, it can be found that the second-order partial derivative of logarithmic stress with respect to logarithmic strain rate ($\partial^{2}ln\sigma /\partial ({ln\dot{\varepsilon })}^{2}$) is relatively small at all strain levels (as shown in Figure 10c, Figure 11c and Figure 12c), while the third-order partial derivative ($\partial^{3}ln\sigma /\partial ({ln\dot{\varepsilon })}^{3}$) is also relatively small (as shown in Figure 10d, Figure 11d and Figure 12d). To make the predicted values more accurately describe the experimental values, the relatively small third-order partial derivatives are also taken into account. Therefore, the relationship between logarithmic stress and logarithmic strain rate can be described by a cubic function.

### 4.2. Establishment of the New Constitutive Model

Based on the previous analysis, the deformation of the constitutive equation with two variables can be expressed by the following formula:

(13) $$\ln\sigma =f(ln\dot{\varepsilon },T)$$

Then, using the Taylor series expansion and more accurately considering the coupling effect of temperature and strain rate, a new constitutive model is proposed, as shown in the following Formula (14):

(14) $$\begin{array}{cc} f(\ln\dot{\varepsilon },T) & =f(0,0)+{\left(\frac{\partial f}{\partial ln\dot{\varepsilon }}\right)}_{\left(0,0\right)}\ln\dot{\varepsilon }+{\left(\frac{\partial f}{\partial T}\right)}_{\left(0,0\right)}T+\frac{1}{2!}{\left(\frac{\partial^{2}f}{\partial (ln\dot{\varepsilon }{)}^{2}}\right)}_{\left(0,0\right)}(\ln\dot{\varepsilon }{)}^{2}\\ & +\frac{1}{2!}(\frac{\partial^{2}f}{\partial T^{2}}{)}_{\left(0,0\right)}T^{2}+(\frac{\partial^{2}f}{\partial ln\dot{\varepsilon }\partial T}{)}_{\left(0,0\right)}\ln\dot{\varepsilon }T+\frac{1}{3!}(\frac{\partial^{3}f}{\partial (ln\dot{\varepsilon }{)}^{3}}{)}_{\left(0,0\right)}(\ln\dot{\varepsilon }{)}^{3}\\ & +{\left(\frac{\partial^{3}f}{\partial (ln\dot{\varepsilon }{)}^{2}\partial T}\right)}_{\left(0,0\right)}(\ln\dot{\varepsilon }{)}^{2}T+{\left(\frac{\partial^{3}f}{\partial ln\dot{\varepsilon }\partial T^{2}}\right)}_{\left(0,0\right)}\ln\dot{\varepsilon }T\\ & +{\left(\frac{\partial^{4}f}{\partial (ln\dot{\varepsilon }{)}^{3}\partial T}\right)}_{\left(0,0\right)}(\ln\dot{\varepsilon }{)}^{3}T+{\left(\frac{\partial^{4}f}{\partial ln\dot{\varepsilon }\partial T^{3}}\right)}_{\left(0,0\right)}(\ln\dot{\varepsilon })T^{3}\\ & +{\left(\frac{\partial^{4}f}{\partial (ln\dot{\varepsilon }{)}^{2}\partial T^{2}}\right)}_{\left(0,0\right)}(\ln\dot{\varepsilon }{)}^{2}T^{2} \end{array}$$

Further simplifying Formula (15):

(15) $$\begin{array}{c} \ln\sigma =k_{1}+k_{2}\ln\dot{\varepsilon }+k_{3}T+k_{4}(\ln\dot{\varepsilon }{)}^{2}+k_{5}\ln\dot{\varepsilon }T+k_{6}T^{2}+k_{7}(\ln\dot{\varepsilon }{)}^{3}+\\ {k_{8}(ln\dot{\varepsilon }{)}^{2}T+k}_{9}\ln\dot{\varepsilon }T^{2}+{k_{10}(ln\dot{\varepsilon }{)}^{3}T+k}_{11}\ln\dot{\varepsilon }T^{3}+k_{12}(\ln\dot{\varepsilon }{)}^{2}T^{2} \end{array}$$

where $k_{1}$, $k_{2}$, $k_{3}$, $k_{4}$, $k_{5}{,\;k}_{6}$, $k_{7}$, $k_{8}{,\;k}_{9}$, $k_{10}$, $k_{11}$, and $k_{12}$ are material parameters. The constant term $k_{1}$ represents the reference value of stress with respect to the strain rate and temperature. The first-order term $k_{2}ln\dot{\varepsilon }$ represents the first-order response of stress to strain rate, describing the linear relationship between stress and logarithmic strain rate. The first-order term $k_{3}T$ represents the first-order response of logarithmic stress to temperature, reflecting the thermal softening effect of the material. The second-order term $k_{4}{\left(ln\dot{\varepsilon }\right)}^{2}$ represents the nonlinear change in logarithmic stress with respect to the logarithmic strain rate, reflecting the nonlinear behavior of the material under strain rate. The cross term $k_{5}Tln\dot{\varepsilon }$ represents the interaction between strain rate and temperature on stress, describing how stress is influenced by both strain rate and temperature, and reflecting the coupling effect of the temperature and strain rate. The second-order term $k_{6}T^{2}$ represents the nonlinear change in logarithmic stress with respect to temperature, reflecting the nonlinear characteristics of the material’s thermal softening behavior. The third-order term $k_{7}{\left(ln\dot{\varepsilon }\right)}^{3}$ represents the third-order response of stress to strain rate, also describing the complex nonlinear relationship between stress and logarithmic strain rate. The term $k_{8}{\left(ln\dot{\varepsilon }\right)}^{2}T$ represents the interaction between the second-order term of strain rate and temperature, describing how stress is influenced by the square of logarithmic strain rate and temperature. The term $k_{9}{ln\dot{\varepsilon }T}^{2}$ represents the second-order interaction between strain rate and temperature, describing how stress is influenced by logarithmic strain rate and the square of temperature. The term $k_{10}{\left(ln\dot{\varepsilon }\right)}^{3}T$ represents the interaction between the third-order term of the logarithmic strain rate and the first-order term of temperature, capturing the high-order nonlinear relationship between logarithmic strain rate and temperature. The term $k_{11}{ln\dot{\varepsilon }T}^{3}$ represents the interaction between the first-order term of the logarithmic strain rate and the third-order term of temperature. The term $k_{12}{\left(ln\dot{\varepsilon }\right)}^{2}T^{2}$ represents the interaction between the second-order term of the logarithmic strain rate and the second-order term of temperature, reflecting the more complex interaction between these two variables.

**Figure 13:**
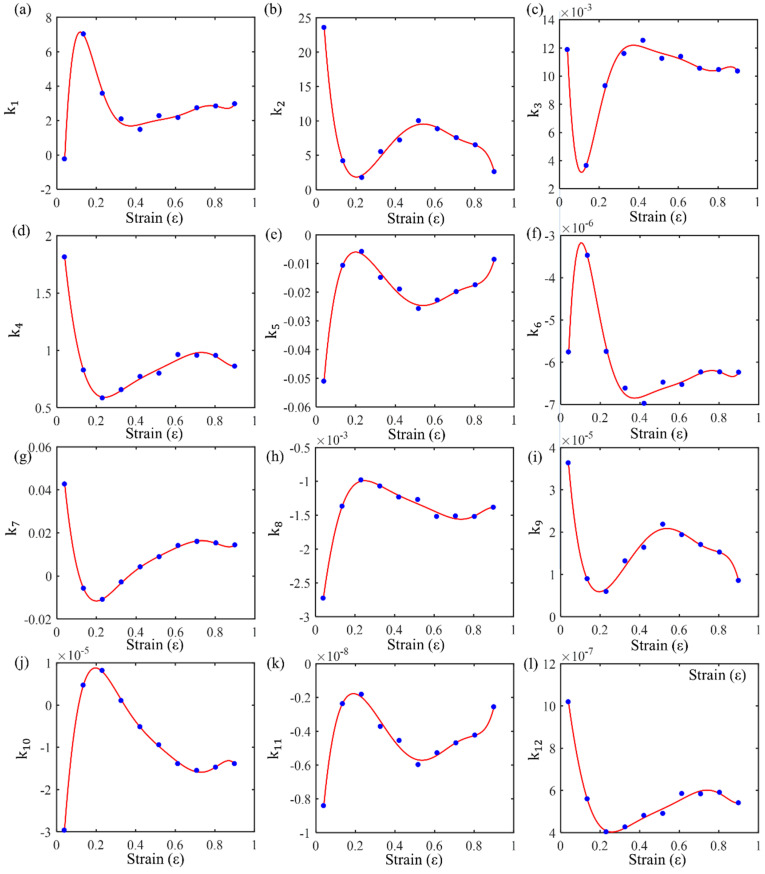
The regression values of material properties at various strain levels and their polynomial fit curves: (**a**) $k_{1}$-ε; (**b**) $k_{2}$-ε; (**c**) $k_{3}$-ε; (**d**) $k_{4}$-ε; (**e**) $k_{5}$-ε; (**f**) $k_{6}$-ε; (**g**) $k_{7}$-ε; (**h**) $k_{8}$-ε; (**i**) $k_{9}$-ε; (**j**) $k_{10}$-ε; (**k**) $k_{11}$-ε; and (**l**) $k_{12}$-ε.

Based on the data provided in Table 3, the material parameters in Formula (15) can be obtained using multiple linear regression. Additionally, since different strains have different material parameters, the relationship between material parameters and strain can be fitted using a polynomial. The degree of the polynomial can be determined based on the regression accuracy. In this study, a seventh-degree polynomial was used to fit these material parameters, and the relationship between each material parameter and different strains was obtained, thus incorporating stress, strain, strain rate, and temperature into the new constitutive equation. The coefficients of each term are shown in Table 5, and the polynomial fitting curves of the quadratic model coefficients at different strain levels are shown in Figure 13. For different materials, the number of coefficients and the order of partial derivatives must be determined based on the complexity of their mechanical behavior. The accuracy of the new constitutive model is also ensured by these factors. Additionally, the coefficients must be solved mathematically.

**Figure 14:**
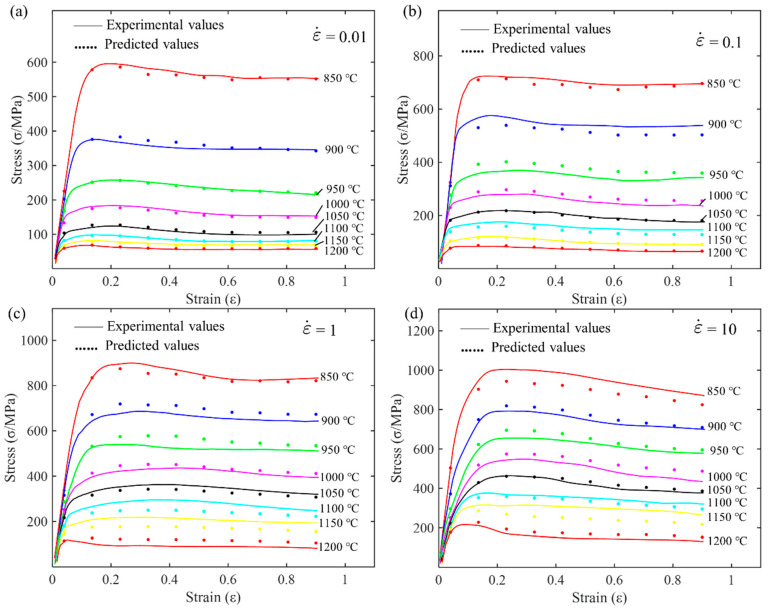
Comparison between predicted rheological stress of the new model and experimental rheological stress at different strain rates: (**a**) 0.01 s^−1^; (**b**) 0.1 s^−1^; (**c**) 1 s^−1^; and (**d**) 10 s^−1^.

By substituting the relationship between the parameters of the new constitutive model for the GH4169 alloy and strain from Table 5 into Formula (15), a complete new constitutive model is obtained. This equation considers the nonlinear relationship between material parameters and strain, allowing the model to more accurately describe the mechanical behavior of the material under different deformation conditions. To evaluate the predictive ability of the newly established constitutive model, the stress predicted by the new model was compared with the experimentally measured data, as shown in Figure 14. In the figure, the solid lines of different colors represent the data obtained from experiments, reflecting the true mechanical behavior of the material under different temperatures and strain rates. The scatter points represent the stress values predicted by the new model under the corresponding conditions. By visually comparing the degree of agreement, the predictive accuracy and reliability of the new model can be qualitatively assessed.

### 4.3. Evaluation of Predictive Performance

To comprehensively evaluate the overall predictive accuracy of the classical model and the new model, a series of standard statistical parameters were introduced, including the correlation coefficient (R), root mean square error (RMSE), sum of squared errors (SSE), and sum of absolute errors (SAE). These parameters quantify the deviation between the model predictions and experimental measurements from different perspectives, providing an objective basis for comparing the performance of different models. The correlation coefficient (R) measures the linear correlation between the model predictions and the experimental values, and its calculation formula is as follows:

(16) $$R=\frac{\sum_{i=1}^{N}\left({\hat{\sigma }}_{i}-\bar{\hat{\sigma }}\right)\left(\sigma_{i}-\bar{\sigma }\right)}{\sqrt{\sum_{i=1}^{N}{\left({\hat{\sigma }}_{i}-\bar{\hat{\sigma }}\right)}^{2}\sum_{i=1}^{N}{\left(\sigma_{i}-\bar{\sigma }\right)}^{2}}}$$

where ${\hat{\sigma }}_{i}$ and $\sigma_{i}$ represent the predicted and experimental values of the ith data point, respectively, $\bar{\hat{\sigma }}$ and $\bar{\sigma }$ represent the mean predicted and experimental values, respectively. The value of R ranges from [−1, 1]. If R is closer to 1, it indicates a stronger positive correlation between the predicted and experimental values. If R is closer to −1, it indicates a stronger negative correlation, while values close to 0 indicate no significant linear correlation between them.

Both the root mean square error (RMSE) and the sum of squared errors (SSE) measure the overall magnitude of the deviation between the model predictions and the experimental values. However, RMSE focuses more on reflecting local deviations, while SSE emphasizes reflecting global deviations. Their calculation formulas are shown in Formulas (17) and (18), respectively:

(17) $$RMSE=\sqrt{\frac{\sum_{i=1}^{N}{\left({\hat{\sigma }}_{i}-\sigma_{i}\right)}^{2}}{N}}$$

(18) $$SSE=\sum_{i=1}^{N}{\left({\hat{\sigma }}_{i}-\sigma_{i}\right)}^{2}$$

where N represents the total number of data points. The smaller the values of RMSE and SSE, the smaller the deviation between the model predictions and the experimental values, indicating higher overall predictive accuracy of the model.

The sum of absolute errors (SAE) calculates the cumulative amount of absolute errors between the model predictions and the experimental values. Its calculation formula is shown in Formula (19):

(19) $$SAE=\sum_{i=1}^{N}\left|{\hat{\sigma }}_{i}-\sigma_{i}\right|$$

This indicator is similar to RMSE and SSE. The smaller the value of SAE, the smaller the absolute deviation between the model predictions and the experimental values, indicating higher overall predictive accuracy of the model.

By calculating the values of these four statistical parameters for both the classical model and the new model, and comparing them, the overall predictive performance of the two models can be quantitatively evaluated. Generally, if the new model achieves better results in these parameters (e.g., R closer to 1, RMSE, SSE, and SAE smaller), it indicates that incorporating the nonlinear relationship between material parameters and strain effectively improves the overall predictive accuracy of the constitutive model. The quantitative comparison of the predictive accuracy of different models is shown in Table 6.

According to the quantitative comparison of the predictive abilities of different models in Table 6, the new model performs the best in all indicators. Its correlation coefficient (R) is 0.9948; the root mean square error (RMSE) is 22.5; the sum of squared errors (SSE) is 16,356; and the sum of absolute errors (SAE) is 5561 MPa. These values indicate that the model has superior fitting and predictive capabilities. The Arrhenius model follows closely, with a correlation coefficient (R) of 0.9888, a root mean square error (RMSE) of 44.1020, a sum of squared errors (SSE) of 19,450, and a sum of absolute errors (SAE) of 8832 MPa. However, its overall performance is slightly inferior to the new model. The HS model shows the poorest performance in all indicators, with a correlation coefficient (R) of 0.9827, a root mean square error (RMSE) of 44.0915, a sum of squared errors (SSE) of 38,881, and a sum of absolute errors (SAE) of 10,877 MPa, indicating its relatively weaker predictive ability.

**Figure 15:**
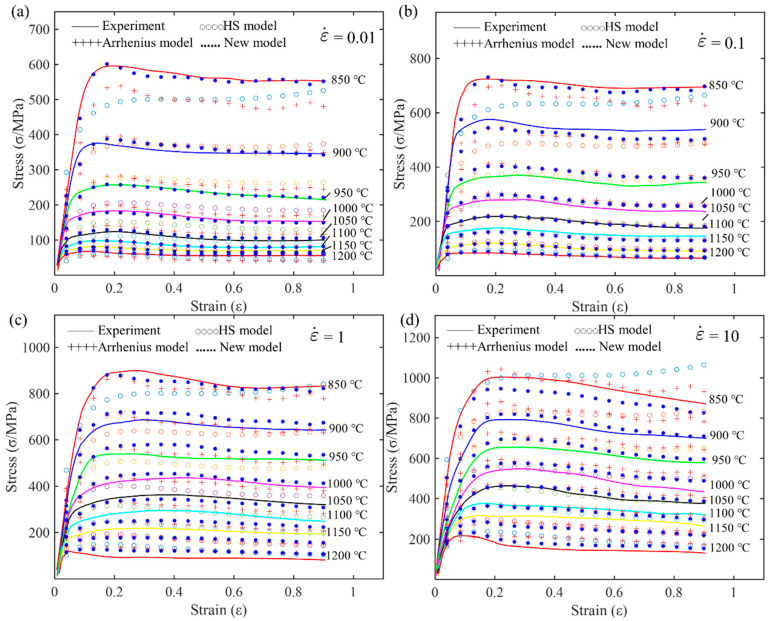
Comparison of predicted flow stress and experimental flow stress at different strain rates for Arrhenius, HS, and new model: (**a**) 0.01 s^−1^; (**b**) 0.1 s^−1^; (**c**) 1 s^−1^; and (**d**) 10 s^−1^.

Figure 15 shows the relationship between the predicted values of different models and the experimental data. The solid line represents the experimental data, hollow circles represent the predictions of the HS model, plus signs represent the predictions of the Arrhenius model, and solid dots represent the predictions of the new model. By analyzing Figure 15a–d, it can be seen that under different strain rates and temperatures, the new model has the best fitting effect, accurately predicting the stress–strain relationship of the material under various conditions. The Arrhenius model follows, fitting the experimental data well under most conditions but performing slightly worse than the new model under high temperatures and high strain rates. The HS model has relatively poor predictive performance, especially under high temperature and high strain rate conditions, where its predictions deviate significantly from the experimental data. Therefore, the new model shows a clear advantage in predicting material behavior.

**Figure 16:**
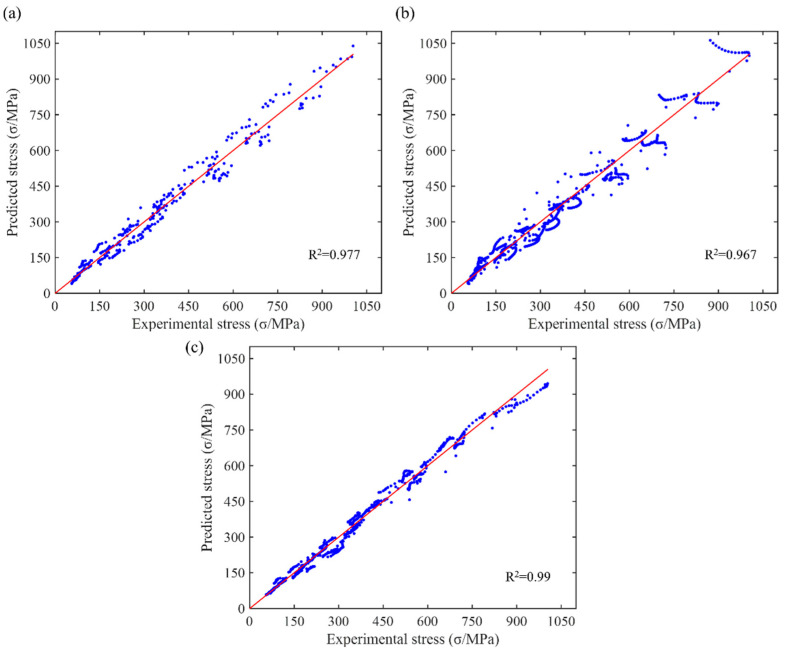
Predictability of flow stress by (**a**) Arrhenius model; (**b**) HS model; (**c**) new model.

Figure 16 shows the predictive ability of different models for flow stress. The coefficient of determination $R^{2}$ for the Arrhenius model is 0.977; for the HS model, it is 0.967; and for the new model, it is 0.99. Combined with the previous analysis results, the new model has the best fitting effect under various strain rate and temperature conditions, accurately predicting the stress–strain relationship of the material and having the highest predictive accuracy. The Arrhenius model follows, with good predictive ability, but it is slightly inferior to the new model. The HS model has the poorest predictive ability, with significant deviations, especially under high-stress conditions. Therefore, the new model demonstrates the highest reliability and accuracy in practical applications. Consequently, this high-precision model, with its mathematical expression, can be integrated into forming simulation software. This integration provides accurate simulations for optimizing existing products or developing new ones, thereby shortening the production cycle.

## 5. Conclusions

In this paper, a new constitutive model based on Taylor series expansions and partial derivatives is proposed. Hot compression tests were conducted at varying strain rates and temperatures to provide the necessary experimental data for model development. The new model significantly improves prediction accuracy without increasing the complexity of material parameters. The predictability of the new model and classical models (Arrhenius and HS models) was comprehensively analyzed, with mathematical proofs provided for the limitations of the classical models. The main conclusions are as follows:

(1) The representative strain levels (low strain ε = 0.136, medium strain ε = 0.518, and high strain ε = 0.9) were selected. By analyzing the partial derivatives of logarithmic stress with respect to temperature and the different orders of logarithmic stress with respect to logarithmic strain rate, it was found that the relationship between logarithmic stress and temperature can be accurately described by a quadratic polynomial function, and the relationship between logarithmic stress and logarithmic strain rate can be effectively described by a cubic polynomial function.

(2) Comparing the error indicators of different models, the new model shows significantly lower root mean square error (RMSE), sum of squared errors (SSE), and sum of absolute errors (SAE). Specifically, the new model achieves an RMSE of 22.5, SSE of 16,356, and SAE of 5561 MPa, outperforming the Arrhenius model (RMSE = 44.1020, SSE = 19,450, SAE = 8832 MPa) and the HS model (RMSE = 44.0915, SSE = 38,881, SAE = 10,877 MPa). This demonstrates its enhanced capability to accurately predict the material’s flow stress under various deformation conditions.

(3) The new constitutive model demonstrates superior prediction accuracy across all tested conditions. It achieves a higher correlation coefficient (R=0.9948) compared to the Arrhenius model (R=0.9888) and the HS model (R=0.9827). This indicates a more accurate representation of the flow stress behavior of the superalloy under various temperatures and strain rates.

In this study, a new constitutive model for a nickel-based superalloy was established based on Taylor series and partial derivatives. By addressing the limitations of traditional models, the new model lays the foundation for future research on constitutive equations and material behavior under extreme conditions. The methodological progress and successful application of the new model highlight its potential applicability to other superalloys and serve as a reference for further research on the deformation behavior of various alloys. Additionally, it provides a research basis for the practical application of other high-temperature materials in production.

## Figures and Tables

**Table 1:** The normal chemical composition of the GH4169 superalloy (wt%).

Ni	Cr	C	Mo	Al	Ti	Nb	Co	Fe
53.25	17.78	0.027	2.98	0.54	1.03	5.50	0.17	Bal.

**Table 2:** High-temperature isothermal compression test parameters.

Heating Temperature (T/°C)	Strain Rate ($\dot{\varepsilon }$/s^−1^)	Heating Rate (°C/s)	Quenching Medium	Amount of Deformation
850	0.01, 0.1, 1, 10	10	water	60%
900				
950				
1000				
1050				
1100				
1150				
1200				

**Table 3:** Stress matrix corresponding to different strain levels (the units of strain rate, temperature, and stress are s^−1^, K, and MPa, respectively).

ε	T	Strain Rate				ε	T	Strain Rate			
		0.01	0.1	1	10			0.01	0.1	1	10
0.040	1123	227.82	329.12	358.83	500.52	0.136	1123	579.01	721.29	833.20	946.92
	1173	195.35	320.85	329.12	383.59		1173	375.33	567.03	643.82	734.94
	1223	167.59	250.16	252.81	287.83		1223	251.24	357.69	526.55	597.42
	1273	145.38	232.08	242.20	246.32		1273	180.57	273.36	396.24	498.63
	1323	97.14	183.29	218.21	229.60		1323	119.93	212.65	330.74	425.92
	1373	80.36	152.73	180.60	200.06		1373	97.87	171.22	265.52	372.52
	1423	68.84	100.02	157.78	196.72		1423	81.78	119.78	207.83	314.70
	1473	59.35	75.99	111.36	174.05		1473	67.50	83.68	101.92	211.57
0.231	1123	594.13	720.76	895.71	1004.20	0.327	1123	582.05	717.33	890.97	999.85
	1173	367.11	569.53	676.67	792.45		1173	357.45	550.91	685.36	788.20
	1223	257.14	367.31	538.91	654.88		1223	251.91	366.95	534.00	654.00
	1273	183.07	279.03	422.19	543.85		1273	177.69	277.96	431.63	546.05
	1323	123.22	218.27	352.52	463.81		1323	115.44	212.85	361.24	457.61
	1373	95.66	175.55	285.40	368.35		1373	89.16	166.83	294.59	364.60
	1423	77.51	118.95	216.78	312.41		1423	73.99	112.19	217.89	314.34
	1473	61.84	82.59	93.27	170.82		1473	58.94	78.92	93.51	158.02
0.422	1123	572.12	707.62	869.63	985.61	0.518	1123	561.43	695.92	845.95	962.25
	1173	351.13	540.84	673.41	770.44		1173	348.24	538.88	661.57	744.37
	1223	243.67	356.47	523.19	643.53		1223	235.68	343.40	518.86	629.54
	1273	168.89	263.34	435.84	529.56		1273	160.98	250.86	431.82	510.61
	1323	107.92	206.57	361.81	436.66		1323	102.21	195.30	354.99	416.44
	1373	83.48	158.99	294.30	357.93		1373	79.20	152.30	288.13	351.29
	1423	71.20	103.57	214.56	307.88		1423	69.57	96.99	209.51	302.36
	1473	56.20	74.16	90.42	149.39		1473	55.75	72.15	89.75	145.00
0.613	1123	556.92	690.43	828.31	937.26	0.709	1123	553.40	691.31	824.04	914.55
	1173	347.41	535.40	653.25	724.84		1173	347.55	534.05	647.91	717.05
	1223	229.25	333.24	517.59	611.25		1223	225.70	332.08	517.82	594.71
	1273	155.64	243.71	421.21	480.04		1273	154.43	238.08	408.95	460.30
	1323	99.15	189.49	346.01	396.89		1323	98.12	182.26	336.02	390.01
	1373	79.07	148.59	277.92	342.63		1373	79.04	145.97	266.15	331.84
	1423	69.35	93.93	203.68	295.81		1423	70.28	92.53	198.67	289.72
	1473	55.83	67.84	89.87	143.58		1473	55.66	65.27	88.54	140.72
0.804	1123	554.37	693.29	828.06	892.76	0.900	1123	553.02	694.59	833.01	872.51
	1173	346.81	535.06	645.02	709.41		1173	345.84	538.56	643.09	700.38
	1223	220.45	338.24	515.93	583.53		1223	215.21	342.81	511.41	578.20
	1273	153.77	238.56	399.79	450.98		1273	153.13	236.39	394.46	434.66
	1323	98.48	177.45	326.81	383.13		1323	100.20	175.78	320.35	377.00
	1373	80.11	146.23	255.73	324.92		1373	82.24	145.82	248.03	320.53
	1423	69.47	91.28	195.26	280.98		1423	70.77	89.87	193.09	263.27
	1473	56.46	64.85	86.52	138.22		1473	56.25	64.82	83.14	131.11

**Table 4:** Multivariate linear regression results of material parameters.

*A*	$m_{1}$	$m_{2}$	$m_{3}$	$m_{4}$	$m_{5}$	$m_{6}$	$m_{7}$	$m_{8}$
7.2 × 10^−12^	−0.0085	0.1208	−0.3011	−0.0235	−0.0021	1.5387	0.00029	6.1773

**Table 5:** Relationship between the coefficients of the new constitutive model for GH4169 alloy and strain.

Coefficient	$\varepsilon^{7}$	$\varepsilon^{6}$	$\varepsilon^{5}$	$\varepsilon^{4}$	$\varepsilon^{3}$	$\varepsilon^{2}$	ε	Constant
$k_{1}$	8447.5693	−31,591.8841	48,563.8604	−39,422.1289	17,971.4331	−4461.1277	519.3204	−14.9232
$k_{2}$	−7703.123	25,570.9945	−35,539.5158	27,469.5274	−13,189.5779	3967.6566	−646.6637	43.8671
$k_{3}$	−11.7467	43.9205	−67.4752	54.6848	−24.8232	6.0934	−0.6870	0.0310
$k_{4}$	240.6360	−703.7447	734.6171	−265.6618	−67.2296	82.7236	−22.5587	2.5885
$k_{5}$	16.6749	−54.3765	74.0737	−56.2616	26.8401	−8.1358	1.3378	−0.0932
$k_{6}$	0.0042	−0.0156	0.0239	−0.0193	0.0087	−0.0021	0.0002	−1.21 × 10^−5^
$k_{7}$	7.4868	−17.3932	8.3725	10.4795	−14.4872	6.8895	−1.3889	0.088
$k_{8}$	−0.4570	1.4129	−1.6479	0.8405	−0.1024	−0.0720	0.0273	−0.0037
$k_{9}$	−0.0122	0.0391	−0.0520	0.0385	−0.0181	0.0055	−0.0009	6.49 × 10^−5^
$k_{10}$	−0.0092	0.0253	−0.0234	0.0046	0.0059	−0.0041	0.0009	−6.05 × 10^−5^
$k_{11}$	3.07 × 10^−6^	−9.65 × 10^−6^	1.25 × 10^−5^	−9.01 × 10^−6^	4.13 × 10^−6^	−1.24 × 10^−6^	2.03 × 10^−7^	−1.48 × 10^−8^
$k_{12}$	0.0002	−0.0006	0.0008	−0.0005	0.0001	7.28 × 10^−6^	−7.67 × 10^−6^	1.31 × 10^−6^

**Table 6:** Quantitative comparison of predictive accuracy of different models.

Indicator	Arrhenius Model	HS Model	New Model
R	0.9888	0.9827	0.9948
RMSE	44.1020	44.0915	22.5
SSE	19,450	38,881	16,356
SAE (MPa)	8832	10,877	5561

## Data Availability

The original contributions presented in the study are included in the article, further inquiries can be directed to the corresponding author.
